# Incidence of and risk factors for acute kidney injury in neonates with congenital diaphragmatic hernia

**DOI:** 10.1007/s00431-025-06513-x

**Published:** 2025-10-10

**Authors:** Andreas Andersson, Bernhard Sedlacek, Jonas Berner, Carmen Mesas Burgos, Johan Mårtensson, Åke Norberg, Urban Fläring

**Affiliations:** 1 Department of Pediatric Perioperative Medicine and Intensive Care, Astrid Lindgren Children’s Hospital, Karolinska University Hospital, Stockholm, Sweden; 2 Department of Physiology and Pharmacology, Karolinska Institutet, Stockholm, Sweden; 3 Ecmo Centre, Department of Pediatric Perioperative Medicine and Intensive Care, Astrid Lindgren Children’s Hospital, Karolinska University Hospital Stockholm, Stockholm, Sweden; 4 Department of Pediatric Surgery, Karolinska University Hospital, Stockholm, Sweden; 5 Department of Women’s and Children’s Health, Karolinska Institute, Stockholm, Sweden; 6 Department of Perioperative Medicine and Intensive Care, Karolinska University Hospital, Stockholm, Sweden; 7 Department of Clinical Science, Intervention and Technology, Karolinska Institutet, Stockholm, Sweden

**Keywords:** Acute kidney injury, Congenital diaphragmatic hernia, Continuous renal replacement therapy, Fluid balance, Neonate

## Abstract

Congenital diaphragmatic hernia (CDH) is a severe congenital malformation requiring surgery in the neonatal period. Neonates with CDH are exposed to several possible risk factors for acute kidney injury (AKI). Still, data on the incidence of and risk factors for AKI in this group of patients are sparse. Our aim was to investigate the AKI incidence and identify modifiable risk factors in a cohort of CDH patients thoroughly characterized in physiological and clinical variables. The primary outcome was the frequency of AKI according to the KDIGO definition. This was a retrospective cohort study including 109 newborn CDH patients with birth weight ≥ 2 kg, GA ≥ 32 weeks, and invasive ventilation initiated within 2 days. Thirty-one patients (28%, 95% CI 21–38) developed AKI, of which 17 (55%) had stage 1, 1 (3%) stage 2, and 13 (42%) stage 3 AKI. Continuous renal replacement therapy was needed in 13 (42%) patients, 12 of these during ECMO treatment. Duration of hyperchloremia prior to AKI was found to be a possibly modifiable risk factor for AKI (*p *= 0.0006). Peak Oxygenation Index (*p* = 0.02) and Vasoactive-Inotropic Score (*p* = 0.0004) were also independently associated with AKI. There was complete resolution of AKI at PICU discharge in all surviving patients. Mortality in included patients was 6/109 (5.5%, 95% CI 2–12).

*Conclusion*: In this cohort, the majority of AKI cases were mild, and surviving patients recovered from AKI before PICU discharge. Almost all cases of severe AKI occurred in ECMO patients. Hyperchloremia could be a modifiable risk factor for the development of AKI in CDH patients.

**Table:** 

**What is Known:** • *Congenital diaphragmatic hernia (CDH) is a congenital malformation with severe cardiorespiratory instability and considerable mortality. The incidence of and risk factors for acute kidney injury (AKI) in CDH patients need further investigation. *
**What is New:** • *The overall rate of AKI was 28 % (95% CI 21-38). The majority of AKI cases were mild, and surviving patients recovered from AKI before PICU discharge.* • *Hyperchloremia was identified as a potentially modifiable risk factor for AKI development.*

**Supplementary Information:**

The online version contains supplementary material available at 10.1007/s00431-025-06513-x.

## Introduction

Critically ill neonates have an increased risk of developing acute kidney injury (AKI) due to low renal blood flow and glomerular filtration rate, most pronounced during their first week of life. AKI can no longer be seen as an incidental finding in critically ill neonates since it is independently associated with an increased mortality and morbidity, including longer duration of mechanical ventilation and length of hospital stay (LOS) [1]. Several risk factors for AKI development have been identified, including the degree of illness, exposure to nephrotoxic drugs, gestational age, and weight. The AKI incidence varies in critically ill neonates due to the number of risk factors and is reported to be 20–70% [1–3]. At present, the Kidney Disease: Improving Global Outcomes (KDIGO) definition for AKI in neonates is by far the most used definition. It was recently validated in a large multicenter study where serum creatinine (SCr) and/or urine output (UOP) were used as definition criteria [1].

Congenital diaphragmatic hernia (CDH) is a rare congenital malformation (1:3000 in live births) characterized by a defect in the diaphragm which allow abdominal organs to herniate into the thorax [4]. Persistent pulmonary hypertension (PPHN) frequently occurs in CDH patients due to combined lung hypoplasia and underdevelopment of the lung vascular tree, hypertrophic pulmonary arteries, and hyperreactivity of these vessels [5]. CDH patients are subjected to several risk factors for AKI, including severe circulatory and respiratory failure and exposure to nephrotoxic drugs.

Fluid overload aggravates pulmonary hypertension and respiratory failure in CDH patients. Strict fluid restriction is a key component of management, but overly restrictive fluid administration might contribute to the development of AKI. The 2015 guidelines from the CDH EURO consortium suggest 40 ml/kg/day of maintenance fluids for the first 24 h. Fluid intake can thereafter increase according to the clinical picture [6]. However, to our knowledge, there are no published data on fluid therapy in CDH patients, and there is a knowledge gap regarding fluid therapy in relation to morbidity, especially with AKI development.

Recent retrospective studies found an incidence of AKI in CDH patients ranging from 38 to 63% [7–9]. However, none of these studies used validated AKI definitions, since only SCr levels were used and UOP data omitted. The AWAKEN trial showed that without measuring UOP, the incidence of AKI would be reduced by approximately one third [1]. This demonstrates that studies using only SCr levels run the risk of underestimating the true incidence of AKI. Moreover, if AKI cases are incorrectly classified as no AKI, the analysis of risk factors for AKI may be flawed. Previous studies have also shown different results with regard to mortality, morbidity, and potential risk factors for AKI [7–9], demonstrating the need for further studies.

The primary aim of this study was to investigate the AKI incidence in a well-defined cohort of CDH patients thoroughly characterized in physiological/clinical variables including fluid balance, UOP, and electrolytes. Secondary aims were to investigate possible modifiable risk factors for AKI, to investigate the prognosis for recovery from AKI, and to describe the relationship between AKI and clinical outcomes.

## Methods

### Study subjects, design, and setting

This was a retrospective observational cohort study conducted at the department of Pediatric Perioperative Medicine and Intensive Care at Karolinska University Hospital, Stockholm, Sweden, a tertiary multidisciplinary referral center for critically ill children and a high-volume referral center for CDH since the centralization of care in 2017. The study period lasted from January 2015 until May 2022. The study procedures were in accordance with the ethical standards of the responsible committee on human experimentation and with the Helsinki Declaration of 1975. The study was approved by the Swedish Ethical Review Authority (AKI in neonates with congenital diaphragmatic hernia, reference No. 2021–05479-01, approval date 20211228). The protocol was registered in ClinicalTrials.gov. Trial number: NCT06050525 registration date 2023-09-06.

Inclusion criteria were newborns with CDH weighing ≥ 2 kg, gestational age ≥ 32 weeks, and invasive ventilation initiated within 2 days of life (DOL). The following exclusion criteria were used: (i) invasive ventilation initiated after more than 2 DOL, since these patients are generally clinically stable and seldom require intensive care, (ii) weight < 2 kg and/or prematurely born < 32 post gestational weeks; these patients were excluded since the care for them is provided in the neonatal intensive care unit, (iii) congenital kidney disease, (iv) death occurring within 2 DOL and/or comorbidity not compatible with life, and (v) late transfer ≥ 3 DOL from another hospital.

### Data collection and definitions

Study data were retrieved from our electronic patient data management systems, Take Care (CGM CompuGroup Medical, Germany) and Centricity Critical Care Clinisoft (GE Healthcare, Stockholm, Sweden) using a standardized collection form. In order to characterize the requirement for inotropic-vasoactive support and degree of respiratory failure, the Vasoactive-inotropic score (VIS) [10] and Oxygenation Index (OI) [11] were used. Retrieved clinical risk factor data included gender, gestational age, antenatal diagnosis of CDH, weight, patch insertion, daily summary of fluid balance (all given fluid and fluid output), PPHN, OI, VIS, SCr, volume of UOP, and plasma chloride concentration. The number of nephrotoxic drugs administered was determined according to previously published definitions [12]. Hyperchloremia was defined as ≥110 mmol/L. Cumulative fluid balance during the first week in the PICU was calculated as: the percentage of fluid balance = (all fluid in − all fluid out)/(birth weight) × 100%. The degree of illness was characterized by Pediatric Index of Mortality-3 (PIM-3) registered on admission. In addition, data on the duration of mechanical ventilation, length of PICU stay, and requirement for extracorporeal membrane oxygenation (ECMO) were also collected. AKI was classified according to the neonatal KDIGO criteria (Table 1) using both SCr concentration and UOP [13, 14] . Both variables were well documented on a daily basis in all patients during the first week in the PICU. The admission SCr was used as a baseline value. In addition, the normal drop in SCr concentration was investigated by comparing the admission value and the value at the last PICU day. An abnormal SCr drop was determined as a SCr above the 97.5 percentile at the patient’s last PICU day [15].

With regard to risk factors for AKI, our main focus was the time period before AKI was established. Therefore, only patients with complete data available from day 1 were included (*n* = 100) in the regression analysis. Furthermore, in order to determine the impact of risk factors prior to AKI development, the time point of AKI development was established and then used to compare the risk factors between patients with and without AKI.

### Statistics

Normality was assessed using a summary for skewness (–1 to 1 as acceptable limits) and visual inspections of the histograms of all variables. Normally distributed variables are presented as mean and standard deviation (SD) and were analyzed by independent 2-sample *t*-test. Nonparametric data are expressed as medians and interquartile range (IQR) and analyzed using Mann–Whitney *U*-test. To compare dichotomized variables, a two-sided Fisher’s exact test was used. A *p*-value < 0.05 was considered to be statistically significant. The proportion for main outcomes was presented as a percentage and 95% confidence intervals according to Wilson Brown.

Predictors of AKI were analyzed by univariate logistic regression followed by step-wise forward multivariable logistic regression analysis. A decrease in deviance by 3.84 (*P* = 0.05) was necessary for a variable to be included in the final predictive model. GraphPad Prism 9 (GraphPad Software, Boston, Massachusetts USA, www.graphpad.com) and NCSS software (NCSS 2023 Statistical Software (2023). NCSS, LLC. Kaysville, Utah, USA, ncss.com/software/ncss) were used for the statistical analyses.

## Results

### Patient selection

During the study period, 146 newborn patients with CDH were eligible to participate in the study. More than 60% of the patients were referred from hospitals outside Stockholm or from abroad. Twenty patients did not require mechanical ventilation during the first 2 DOL, and seven patients died during the same time span or had a comorbidity not compatible with life. Other reasons for exclusion were prematurity (*n* = 8), congenital kidney disease (*n* = 1), and late transfer from another PICU (*n* = 1). This resulted in a final cohort of 109 included patients.

**Fig. 1:**
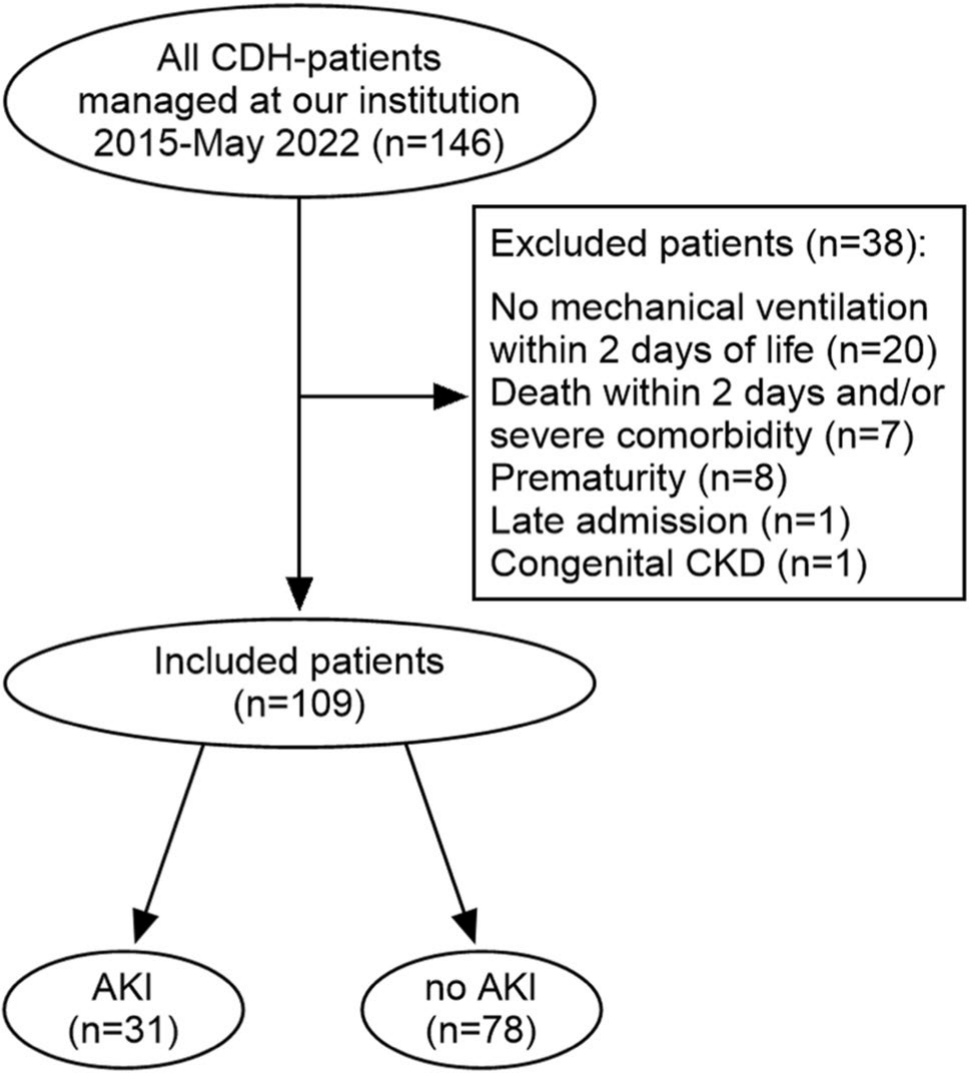
Flowchart showing the selection of patients. CDH, congenital diaphragmatic hernia; AKI,  acute kidney injury

A flow chart is given in Fig. 1.

### AKI development

Thirty-one patients (28%, 95% CI 21–38) developed AKI according to the KDIGO criteria, of which 17 (55%) had stage 1, 1 (3%) had stage 2, and 13 (42%) had stage 3 AKI. All patients were diagnosed with AKI based on an increase in SCr. In five (16%) patients, UOP confirmed the AKI diagnosis. No patients were diagnosed with AKI based only on UOP. The median time-point for AKI development was 3 days (IQR 2–3 days).

AKI was more common in patients requiring ECMO (15 of 24 (62%)) than in patients not requiring ECMO (16 of 85 (19%)), *p* < 0.0001. In addition, AKI was more severe among ECMO patients (stage 1, *n* = 2; stage 2, *n* = 1; stage 3, *n* = 12) as compared to patients not requiring ECMO (stage 1, *n* = 15; stage 2, *n* = 0; stage 3, *n* = 1). Among AKI patients, 13 (42%) received continuous renal replacement therapy (CRRT); 12 of these patients received CRRT during ECMO treatment. All patients receiving CRRT were treated with Continuous Veno-Venous Hemodiafiltration.

Using the KDIGO definition, there was complete resolution of AKI at PICU discharge in all surviving patients. However, in surviving patients with AKI, 7/27 (26%, 95% CI 13–45) had SCr above the 97.5 percentile of the normal decline at the last PICU day, as compared to 4/77 (5%, 95% CI 2–13, *p* = 0.006) in patients with no AKI.

### Mortality

In screened and included patients, the mortality was 14/146 (9.6%, 95% CI 6–15) and 6/109 (5.5%, 95% CI 2–12), respectively. In screened patients, there were five early deaths, including four patients with major pulmonary hypoplasia (including one case of bilateral herniation) and one patient with vascular malformations making ECMO cannulation impossible. Two patients received comfort care due to severe comorbidity not compatible with life (one case of severe chromosomal abnormality and one case of congenital heart disease). Furthermore, one patient with late transfer (14 DOL) also died in the PICU.

Among included patients, the mortality was 4/31 (12.9%, 95% CI 5–29) and 2/78 (2.6%, 95% CI 0.5–9) in AKI and no AKI patients, respectively, *p* = 0.054. All deaths occurred in ECMO patients.

### Characteristics

Patient characteristics are shown in Table 1. CDH was prenatally diagnosed in 64% of the patients, 81% and 56% among patients with and without AKI, respectively (*p* = 0.013). Congenital heart disease occurred in nine patients (8.3%) and other comorbidities were present in 10.1% of patients. ECMO support was required in 24/109 patients (22%). On admission, patients in the AKI group were more severely ill as compared to the no AKI group, PIM-3 score 17 (13–26) and 12 (9.2–16), respectively (*p* = 0.0001). Also, PPHN was more frequent in patients with AKI (75%) as compared to patients without AKI (37%) (*p* = 0.0004). Peak VIS was higher in AKI patients as compared to the no AKI group (27 (IQR 14–35) vs 11 (IQR 5.8–17), *p* < 0.0001). The AKI group also had a significantly higher OI, 38 (IQR 14–59) vs 10 (IQR 6.0–18), *p* < 0.0001. PPHN, OI, and VIS were most pronounced during the first 3 days in the PICU. The median number of nephrotoxic drugs (*p* < 0.0001) and the median duration of hyperchloremia (*p* < 0.0001) were higher in AKI patients.

**Table 1:** Patient characteristics in neonates with congenital diaphragmatic hernia by acute kidney injury

Variable	All patients (*n* = 109)	AKI (*n* = 31)	No AKI (*n* = 78)	*p*
Gestational age (week), Mean (SD)	38 (1.8)	37.6 (2.0)	38.2 (1.7)	0.18
Prematurity, *n* (%)	20 (18)	8 (26)	12 (15)	0.27
Birthweight (g), mean (SD)	3102 (472)	2992 (482)	3146 (464)	0.13
Gender male, *n* (%)	80 (73)	19 (61)	61 (78)	0.09
CHD, *n* (%)	9 (8.3)	3 (9.7)	6 (7.7)	0.71
Other comorbidity, *n* (%)	11 (10.1)	5 (16.1)	6 (7.7)	0.29
Left-sided CDH, *n* (%)	83 (76)	22 (71)	61 (78)	0.46
Patch, *n* (%)	73 (67)	27 (87)	46 (59)	0.006
Length of stay in the PICU (days), median (IQR)	18.5 (10–28)	26 (15–38)	13 (8–24)	0.0001
PIM-3 (%), median (IQR)	14 (9–18)	17 (13–26)	12 (9–16)	0.0002
Peak Vaso-Inotropic Score, median (IQR)	13 (6–22)	27 (13–35)	10 (6–17)	<0.0001
Peak Oxygenation Index, median (IQR)	13 (7–31)	38 (14–59)	10 (6–18)	<0.0001
Mechanical ventilation (days), median (IQR)	12 (7–21)	21 (12–33)	9 (6–18)	<0.0001
PPHN, *n* (%)	52 (48)	23 (74)	29 (37)	0.0006
ECMO, *n* (%)	24 (22)	15 (48)	9 (13)	<0.0001
Mortality, *n* (%)	6 (6)	4 (13)	2 (3)	0.05
Age of surgery (days), median (IQR)	4 (3–5)	4 (4–6)	4 (3–5)	0.05
Antenatally confirmed CDH, *n* (%)	70 (64)	25 (81)	44 (56)	0.013
Intubated within 6 h, *n* (%)	100 (92)	31 (100)	69 (88)	0.06
Peak Furosemide dose during first week (mg/kg/day), mean (SD)	4.9 (5.6)	5.0 (6.1)	4.9 (5.4)	0.94
Duration (days) of hyperchloremia during the first 3 days, median (IQR)	2.1 (1.2–2.5)	2.5 (1.9–2.9)	2 (0.8–2.2)	0.0004

*AKI* acute kidney injury, *CDH* congenital diaphragmatic hernia, *CHD* congenital heart disease, *ECMO* extracorporeal membrane oxygenation, *IQR* interquartile range, *PICU* pediatric intensive care unit, *PIM-3* pediatric index of mortality score-3, *SD* standard deviation

*p*-values between patients with and without AKI were calculated by independent 2-sample *t*-test or Mann–Whitney *U*-test

The median day of surgery was day 4 in both groups. Patch requirement was more frequent in AKI patients (87%) compared to no AKI patients (59%), *p* = 0.006. AKI was associated with a longer duration of mechanical ventilation (*p* = 0.0001) and a longer PICU stay (*p* < 0.0001).

### Fluid balance

**Fig. 2:**
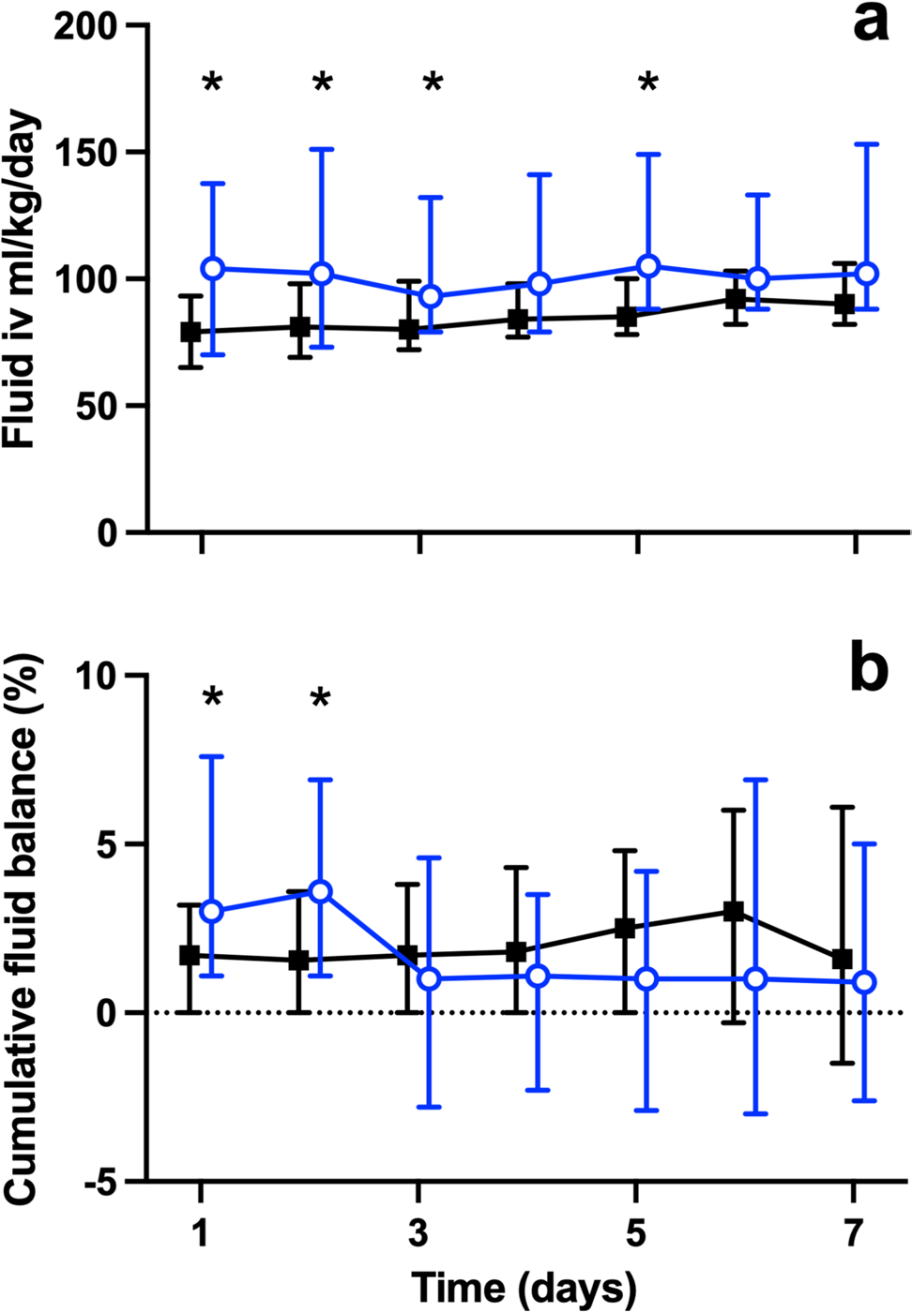
Total daily given fluid (**a**) and cumulative fluid balance (**b**) in patients with and without AKI during the first week in the PICU. The open dots depict patients with AKI and closed squares patients with no AKI. Adjusted *p* values (*p < 0.05) by Mann-Whitney *U* test between AKI and no-AKI at each time point were performed with Bonferroni correction for multiple testing. AKI, acute kidney injury; PICU, pediatric intensive care unit

On the admission day, the median administered fluid volume was 82 (IQR 67–103) ml/kg, with AKI patients receiving more fluid as compared to no AKI patients (104 (IQR 70–138) vs 79 (IQR 65–93) ml/kg, *p* = 0.006). AKI patients received more fluid in 4 out of 7 days during the first week (Fig. 2a). The median daily fluid volume during the first week was 102 and 84 ml/kg/day in AKI and no AKI patients, respectively. The median fluid balance in % of body weight for day 1 in AKI and no AKI patients was 3.0 (IQR 1.1–7.6) and 1.7 (IQR 0–3.2), respectively (*p* < 0.0036). At day 7, there was no difference in cumulative fluid balance between the groups (2.3% ± 7.0 vs 2.4% ± 5.9) (Fig. 2b). Peak positive fluid balance was significantly higher in the AKI group, 9.1% ± 8.1 vs 5.8% ± 4.4 (*p* < 0.008). Most patients (100 out of 109) received furosemide therapy, and the mean peak dose in AKI and no AKI patients during the first week was 5.0 ± 6.1 and 4.9 ± 5.4 mg/kg/day, respectively.

### ECMO vs no ECMO patients

In general, ECMO patients had a more pronounced degree of illness as compared to no ECMO patients: PIM-3 (33.2 ± 25.6 vs 12.1 ± 4.1, *p* < 0.001), VIS (29.4 ± 24.5 vs 13.6 ± 11.7, *p* < 0.001), OI (59.5 ± 34.1 vs 13.3 ± 10.3, *p* < 0.001), and PPHN (100% vs 32.9%, *p* < 0.001). ECMO patients were more exposed to more nephrotoxic drugs (3.2 ± 1.2 vs 0.4 ± 0.7, *p* < 0.001) and had a higher peak fluid overload during the first week (13.7% ± 7.1 vs 4.7% ± 3.5, *p* < 0.0001) as compared to patients not requiring ECMO (eFig 1a). On the admission day, ECMO patients received 139 (IQR 170–119) ml/kg compared to 78 (IQR 93–65) ml/kg for patients not receiving ECMO, *p* < 0.0001. The total infused daily fluid volume was higher in ECMO patients for every day (eFig 1b).

### Regression analysis of risk factors for AKI

On univariate analysis, several proposed risk factors (9/11) were significantly associated with AKI (Table 2). In order to minimize covariation and considering the relatively low number of events, the variables were stepwise investigated in the multivariate analysis. In the final multivariate analysis, the duration of hyperchloremia (*p* = 0.0006), peak OI (*p* = 0.02), and peak VIS (*p* = 0.0004) during the time frame prior to AKI diagnosis remained significantly associated with AKI (Table 3).

**Table 2:** Predictors of acute kidney injury in children with congenital diaphragmatic hernia by univariate logistic regression (*n* = 100)

Variable	Odds ratio	*p*
Oxygenation index on admission (per point)	1.06 (1.03–1.09)	<0.0001
Numbers of nephrotoxic drugs prior to AKI (per drug)	2.89 (1.66–5.00)	0.0001
PIM-3 (per %)	1.11 (1.02–1.22)	0.0002
PPHN (yes)	4.41 (1.69–11.5)	0.002
Patch (yes)	4.19 (1.31–13.4)	0.008
Side (left)	0.52 (0.19–1.39)	0.20
Vaso-inotropic score prior to AKI (per point)	1.08 (1.04–1.13)	<0.0001
Duration of hyperchloremia prior to AKI (per day)	3.29 (1.55–6.93)	0.0002
Peak Fluid overload prior to AKI (per %)	1.16 (1.04–1.30)	0.005
Weight (per g)	0.999 (0.997–0.999)	0.02
Gestational age (per week)	0.79 (0.62–1.02)	0.07

*AKI* acute kidney injury, *PIM-3* pediatric index of mortality score-3

**Table 3:** Predictors of acute kidney injury (AKI) in children with congenital diaphragmatic hernia by step-wise forward multivariable logistic regression analysis (*n* = 100)

Variable	Odds ratio	*p*
Duration of hyperchloremia prior to AKI (per day)	3.75 (1.55–9.05)	0.0006
Vaso-inotropic score prior to AKI (per point)	1.09 (1.03–1.15)	0.0004
Oxygenation index on admission (per point)	1.04 (1.03–1.07)	0.02

## Discussion

In this retrospective single-center study, the overall rate of AKI in CDH patients was 28%. Hyperchloremia was identified as an independent and potentially modifiable risk factor for the development of AKI. The majority (55%) of AKI cases were mild, and almost all cases of severe AKI occurred in ECMO patients. According to KDIGO criteria, there was resolution of AKI in all surviving patients.

The incidence of AKI (28%) in our study was low as compared to previous studies finding AKI in 38–63% of CDH patients [7–9]. Eight patients were excluded due to early mortality, and likely these patients also suffered from AKI. On the other hand, we also excluded 20 cases of less severe CDH with an uncomplicated clinical course and no organ failures. PICU mortality was low, 5.5% in included patients and 9.6% in the entire cohort. Recent data from the CDH study group including over 5000 CDH patients found a higher hospital mortality of 28.2% [16]. Our center is a tertiary high-volume center with in-house ECMO and strict protocols for CDH management; possibly this could have contributed to the low mortality observed. Other factors that could influence mortality are local case-mix and surgical management.

Previous studies have published conflicting results of the association between AKI and mortality in CDH patients [7–9]. Since mortality in the included patients was very low, 5.5%, firm conclusions regarding the association between AKI and mortality could not be drawn for our data. However, AKI was associated with a longer duration of mechanical ventilation and PICU stay.

The majority of AKI cases (55%) were mild, KDIGO stage 1. The significance of KDIGO stage 1 AKI for relevant clinical outcomes remains unclear [17]. Severe AKI (stage 2 and 3) occurred almost exclusively (13/14 cases) in patients on ECMO, and 54% of ECMO patients were diagnosed with severe AKI. This is in line with previous data finding that 49% of CDH patients on ECMO suffered from severe AKI [18] defined as Failure using the RIFLE criteria [19]. The increased risk of severe AKI in ECMO patients is likely secondary to greater severity of illness. Other factors possibly contributing to AKI development in ECMO patients include activation of systemic inflammation, hemolysis, renal microthrombosis, reperfusion injury and non-pulsatile blood-flow [20]. In our study, all KDIGO stage 3 patients on ECMO received CRRT. Indications for CRRT initiation can differ between ECMO and non-ECMO patients. In ECMO therapy, volume overload is a frequent problem and the most common indication for CRRT initiation [21, 22], potentially leading to earlier initiation of CRRT.

Hyperchloremia with increasing chloride load to the macula densa in the nephron can cause renal vasoconstriction and reduced glomerular filtration rate through the tubular glomerular feedback mechanism [23]. Since AKI in itself can lead to hyperchloremia, we investigated the effects of hyperchloremia before AKI onset to avoid this potential confounder. Interestingly, the duration of hyperchloremia before the development of AKI was identified as an independent risk factor for the development of AKI. Our results are in line with recent data demonstrating an increased risk for AKI in critically ill children with hyperchloremia [24]. Moreover, previous studies have demonstrated an association between hyperchloremia and mortality in PICU patients [25, 26]. In the neonatal population, previous data regarding the causes and effects of hyperchloremia is very limited, but a high chloride load in combination with immature kidneys could make severely ill neonates particularly vulnerable to hyperchloremia. Critically ill children receive a significant chloride load through fluid creep (fluids administered as a vehicle for intravenous drugs, to provide patency of catheters, and for flushes) [27]. In order to reduce the chloride load and possibly prevent the development of hyperchloremia, medications can be dissolved in glucose instead of in saline when possible, and for resuscitation, saline can be replaced by resuscitation fluids with lower chloride content. Although avoiding hyperchloremia could potentially reduce the incidence of AKI in CDH patients, the role of hyperchloremia in the development of AKI is still not fully understood. Moreover, given the retrospective nature of our data, the possibility of hyperchloremia being a marker of increased severity of illness cannot be ruled out, and prospective studies on the topic are needed.

Peak OI and VIS were also identified as independent risk factors for AKI. It is reasonable to assume that these factors reflect renal effects of the hypoxic respiratory failure and severe hemodynamic effects related to CDH pathophysiology. This is further supported by the fact that almost all cases of severe AKI occurred in ECMO patients. OI has previously been demonstrated to be an independent risk factor for AKI in CDH [7].

In PICU patients, fluid overload has been associated with morbidity as well as mortality [28–30]. CDH patients are considered to be particularly vulnerable to fluid overload, and recommendations for management include meticulous fluid restriction. The CDH euro consortium recommends 40 ml/kg of fluids with additional volume top-up in the case of inadequate tissue perfusion [6]. Our local protocol for CDH management includes maintenance fluid administration of 60 ml/kg/d in the first 24 h for patients not receiving ECMO treatment; thereafter, fluid intake can be carefully increased if the clinical picture allows for it. Initially, glucose-containing solutions are used for maintenance fluid, and parenteral nutrition is started after five days. More fluid than recommended in our institutional protocol was administered since patients not on ECMO required 78 ml/kg/d in the first 24 h. A possible explanation for this is that CDH patients are often severely ill with cardiorespiratory instability, and fluid from drug infusions and iv flush can amount to a significant volume. Besides restricting fluid intake, achieving adequate diuresis is also of utmost importance when trying to avoid fluid overload. Our institutional protocol includes strict fluid balance control with early use of diuretic therapy. The fact that our AKI incidence was relatively low compared to previous studies and that most cases were AKI grade 1 indicates that aggressive diuretic therapy to avoid fluid overload does not have negative renal consequences in CDH patients.

At PICU discharge there was complete AKI resolution in all surviving patients using the KDIGO definition. This indicates that most CDH patients recover from AKI in the short perspective, but the long-term effects of AKI in the neonatal period remain to be clarified. Moreover, one problem with the neonatal KDIGO definition is that interference from the maternal SCr complicates the determination of a stable baseline SCr [31]. Considering published reference values for serum SCr in neonates, 7/27 (26%) of surviving patients with AKI and 4/77 (5%) of patients with no AKI had SCr above the 97.5 percentile at the last PICU day. Failure to decrease SCr levels at the expected rate during the first week of life has been associated with adverse outcomes in patients with hypoxic ischemic encephalopathy [32]. The possibility of remaining renal damage in neonates where the expected drop in SCr is absent cannot be disregarded and warrants further investigations.

Previous studies have demonstrated the importance of including the UOP criteria when investigating AKI in neonates and children [13; 17]. This is the first study on CDH patients using the full KDIGO AKI definition with reliable data on UOP as well as SCr levels. All 31 cases of AKI were diagnosed based on the SCr criteria, but only 4 of these patients also fulfilled the UOP criteria. No patient was diagnosed with AKI based solely on UOP. This implies that AKI in CDH patients is mainly a non-oliguric renal failure. Our results differ from the AWAKEN study, where 30% of AKI cases with both SCr levels and UOP available were diagnosed based only on low UOP [13]. A possible explanation for this is our aggressive use of diuretics in order to avoid positive fluid balances.

### Strengths and limitations

The inclusion of consecutive CDH patients and our robust database allowing us to use the full KDIGO definition are the main strengths of the study. One limitation is the retrospective, single-center design. Moreover, even though we included both the SCr and UOP criteria of the KDIGO definition, there are still methodological challenges in diagnosing neonatal AKI, as discussed above.

## Conclusions

In this retrospective study from a high-volume referral center, the majority of AKI cases were mild, and all surviving CDH patients had recovered renal function at PICU discharge according to the KDIGO criteria. Hyperchloremia before AKI diagnosis was found to be a potentially modifiable risk factor for AKI. Other independent risk factors were OI and VIS score. In this group of patients, using the KDIGO UOP criteria did not add any new cases of AKI, indicating that the AKI in CDH is mainly non-oliguric.

## Supplementary Information

Below is the link to the electronic supplementary material.

ESM 1 Supplementary Material 1 (DOCX 240 KB)

## Data Availability

Datasets are available from the corresponding author upon reasonable request.

## Abbreviations

AKI: Acute kidney injury
CDH: Congenital diaphragmatic hernia
DOL: Days of life
ECMO: Extra corporeal membrane oxygenation
IQR: Interquartile range
KDIGO: Kidney Disease: Improving Global Outcomes
LOS: Length of stay
OI: Oxygenation Index
PIM-3: Pediatric Index of Mortality-3
PPHN: Persistent pulmonary hypertension
SCr: Serum creatinine
SD: Standard deviation
UOP: Urine output
VIS: Vasoactive-Inotropic Score
