# Supplementary material for: Incidence of and risk factors for acute kidney injury in neonates with congenital diaphragmatic hernia
Source: Eur J Pediatr. 2025 Oct 10;184(11):673. doi: 10.1007/s00431-025-06513-x (PMC12513905; doi:10.1007/s00431-025-06513-x)
Supplement: Supplementary file 1 — Supplementary Material 1 (DOCX 240 KB) [file 431_2025_6513_MOESM1_ESM.docx]

**
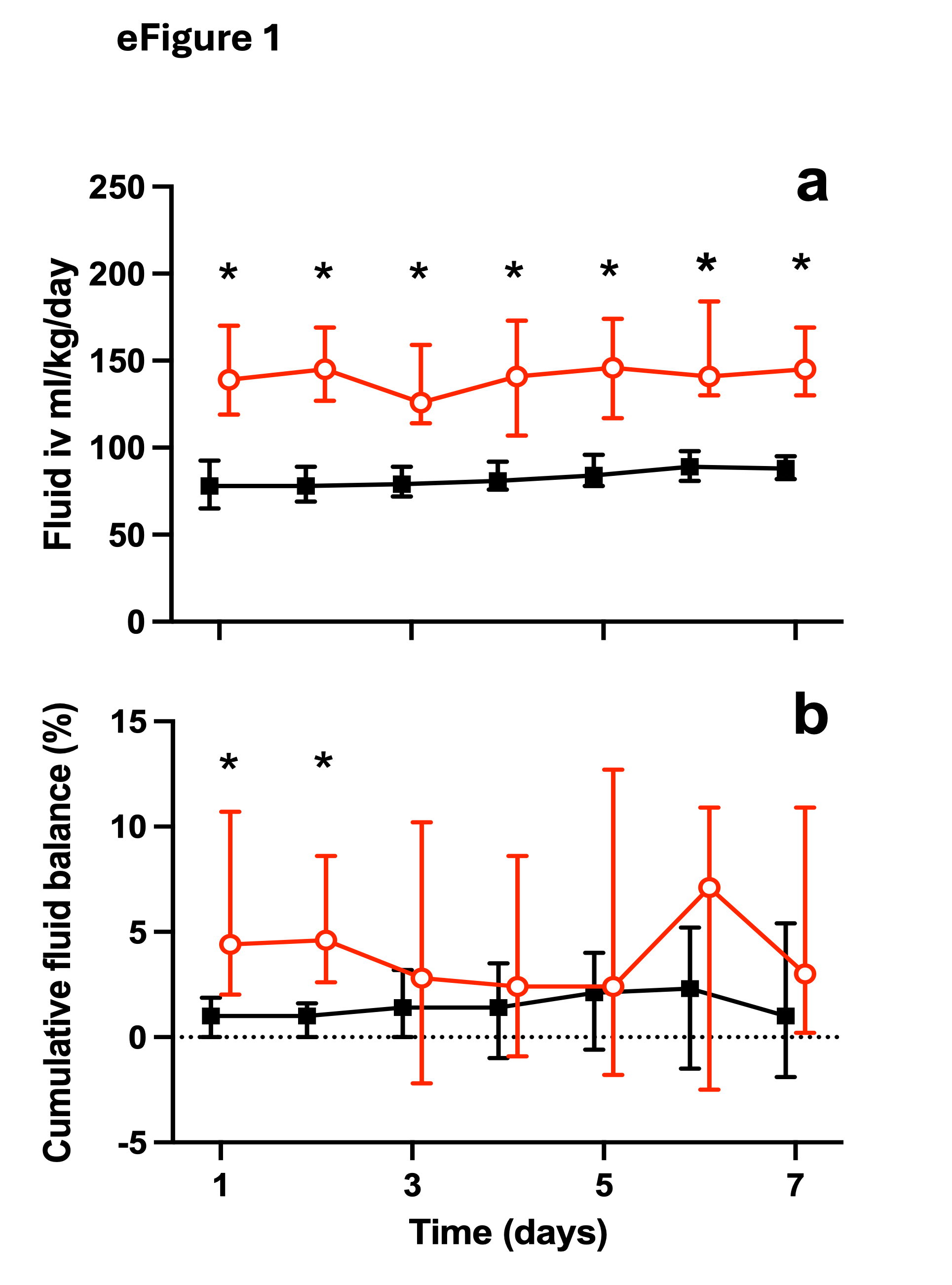
**

**eFig. 1**

Total daily given fluid (a) and cumulative fluid balance (b) in ECMO patients and no ECMO patients. The open dots depict ECMO patients and closed squares no ECMO patients. Adjusted P values (*p < 0.05 ) by Mann-Whitney U test between ECMO and no ECMO patients at each time point, was performed with Bonferroni correction for multiple testing.

**eTable 1. Neonatal AKI KDIGO staging and definition.**

| **AKI stage** | **Serum creatinine (SCr)** | **Urine output over 24 hours** |
| --- | --- | --- |
| 0 | No change in SCr or SCr rise < 0.3 mg/dL | > 1 mL/kg/hour |
| 1 | SCr rise ≥ 0.3 mg/dL within 48 hours or  SCr rise ≥1.5–1.9 X reference SCr* within 7 days | > 0.5 and ≤ 1 mL/kg/hour |
| 2 | SCr rise ≥ 2–2.9 X reference SCr* | > 0.3 and ≤ 0.5 mL/kg/hour |
| 3 | SCr rise ≥ 3 X reference SCr* or  SCr ≥ 2.5 mg/dL** or  Receipt of dialysis | ≤ 0.3 mL/kg/hour |

*Reference SCr is the lowest prior SCr measurement.
